# The Impact of Social Factors on Human Immunodeficiency Virus and Hepatitis C Virus Co-Infection in a Minority Region of Si-Chuan, the People's Republic of China: A Population-Based Survey and Testing Study

**DOI:** 10.1371/journal.pone.0101241

**Published:** 2014-07-02

**Authors:** Caiting Dong, Z. Jennifer Huang, Maria C. Martin, Jun Huang, Honglu Liu, Bin Deng, Wenhong Lai, Li Liu, Yihui Yang, Ying Hu, Guangming Qin, Linglin Zhang, Zhibin Song, Daying Wei, Lei Nan, Qixing Wang, Hongxia Deng, Jianxun Zhang, Frank Y. Wong, Wen Yang

**Affiliations:** 1 Department of HIV Control and Prevention, Si-chuan Center for Disease Control and Prevention, Chengdu, China; 2 Department of International Health, Georgetown University, Washington DC, United States of America; 3 Department of Pediatrics - Adolescent & Young Adult Medicine, University of California San Francisco, San Francisco, California, United States of America; 4 Department of HIV Control and Prevention, Liangshan Prefecture Health Bureau, Xichang, Si-chuan, China; 5 Department of HIV Control and Prevention, Lianshan Center for Disease Control and Prevention, Xichang, Si-chuan, China; 6 Department of HIV Control and Prevention, Butuo Health Bureau, Butuo, Si-chuan, China; 7 Department of Behavioral Sciences and Health Education, Emory University, Atlanta, Georgia, United States of America; Temple University School of Medicine, United States of America

## Abstract

**Background:**

While many human immunodeficiency virus (HIV) studies have been performed in Liangshan, most were focused only on HIV infection and based on a sampling survey. In order to fully understand HIV and hepatitis C virus (HCV) prevalence and related risk factors in this region, this study implemented in 2009, included a survey, physical examination, HIV and HCV test in two towns.

**Methods:**

All residents in two towns of the Butuo county were provided a physical examination and blood tests for HIV and HCV, and then followed by an interview for questionnaire.

**Results:**

In total, 10,104 residents (92.4%) were enrolled and 9,179 blood samples were collected for HIV and HCV testing, 6,072 were from individuals >14 years old. The rates of HIV, HCV, and HIV/HCV co-infection were 11.4%, 14.0%, and 7.7%, respectively for >14-year-old residents. The 25–34 yr age group had the highest prevalence of HIV, HCV, and HIV/HCV co-infections, reaching 24.4%, 26.2% and 16.0%, respectively. Overall, males had a much higher prevalence of all infections than females (HIV: 16.3% vs. 6.8%, HCV: 24.6% vs. 3.9%, HIV/HCV co-infected: 14.7% vs. 1.1%, respectively; *P* = 0.000). Approximately half of intravenous drug users tested positive for HIV (48.7%) and 68.4% tested positive for HCV. Logistic regression analysis showed that five factors were significantly associated with HIV and HCV infection: gender (odds ratio [OR]  = 5.8), education (OR = 2.29); occupation (student as reference; farmer: OR = 5.02, migrant worker: OR = 6.12); drug abuse (OR = 18.0); and multiple sexual partners (OR = 2.92). Knowledge of HIV was not associated with infection.

**Conclusion:**

HIV and HCV prevalence in the Liangshan region is very serious and drug use, multiple sexual partners, and low education levels were the three main risk factors. The government should focus on improving education and personal health awareness while enhancing drug control programs.

## Introduction

By the end of 2010, the Chinese government estimated that 780,000 people were infected with human immunodeficiency virus/acquired immune deficiency syndrome (HIV/AIDS) in the People's Republic of China [1]. In Si-chuan province, which is located in the southwest of China, 48,357 cases of HIV infection were reported by the end of 2011 [2], which is among the five Chinese provinces with the highest HIV prevalences [1]. More than half of the HIV/AIDS patients (53%) in Si-chuan province live in the Liangshan Yi autonomous prefecture, which shares borders with Yunnan and is in close proximity to the Golden Triangle region where large amounts of heroin are manufactured and trafficked. Almost half of Liangshan's population of 4.73 million are members of the Yi minority and live in rural areas [3]. Rugged mountainous terrain and the sparsely scattered rural populations have hindered the economic development of this region, and Liangshan remains one of the poorest areas in the People's Republic of China.

Sociological research has linked the high HIV prevalence in this area to a range of historical and cultural factors, including poverty, low education, and indigenous customs [4], [5]. For more than 10 years since the first HIV infection was documented in 1995, the Liangshan AIDS epidemic has been concentrated among intravenous drug users (IDUs) [6]–[8]. In recent years, however, the proportion of cases among IDUs has decreased; on the other hand, heterosexual transmission has begun to rise [2].

Studies performed in other provinces showed that co-infection with the hepatitis C virus (HCV) is common among HIV positive individuals and IDUs because they share the same transmission routes [9]–[11]. Heterosexual transmission of HIV among non-IDU sexual partners was also reported and is currently attracting increasing attention. Co-infection of HIV and HCV can result in liver cirrhosis and can increase HIV morbidity and mortality [12], [13]. Understanding the transmission routes of both HIV and HCV has significant indications for implementing anti-retroviral therapy.

In 2008, a large scale HIV screening was conducted in Butuo county.The target population was local residents aged from 14 to 60 years old, and the size was 84 357, but the study only got 30 111 samples, the response rate was 35.7 percentage. The result showed that HIV epidemic was extremely severe, and HIV prevalence was highest among China. In order to fully understand HIV and hepatitis C virus (HCV) prevalence and related risk factors in this region, a national HIV strategy has been implemented in two towns of Butuo since 2009, conducting population-based surveillance and testing campaigns and implementing the Four Frees and One Care policy [14], which includes free HIV testing and antiretroviral medication for HIV-affected individuals. The population surveillance program provides a unique opportunity to understand the scale and determinants of HIV and HCV co-infection epidemic as well as risk factors for the spread of infectious disease in this remote and isolated region. This study uses baseline data from the population survey and testing data obtained in 2010 from two counties with 99% of its population consisting of the Yi minority group. In this paper, we principally focused on the impact of social factors, including educational status, occupation, drug abuse, knowledge of transmission types and multiple sexual partners. We will assess the associations between HIV and HCV prevalence and will examine patients' socio-demographic characteristics, knowledge of HIV, and drug use status by gender and marital status.

## Methods

### Study setting and sample

The population survey and testing study were conducted in two towns (A and B) of the Butuo district between April 2010 and December 2010. These towns were selected out of 30 towns, which had similar social demographic characters (such as, age structure, education level, constituent ratio of Yi people, economic level, etc.), meanwhile this two towns had high prevalence rate of HIV. To be eligible for the study, participants had to: (1) be a resident of the town or have lived there for more than 3 months at baseline, and (2) give consent to participate in the study.

To improve participant enrollment, the local government and Si-chuan Center for Disease Control (CDC) completed a series of preparation steps with the 11 village chiefs (six in town A and five in town B). The chiefs were first contacted and invited to health education sessions provided by the CDC. Next, the village chiefs were also asked to collect basic demographic information to register all villagers, including name, age, gender, marital status, migrant status, drug use status, and so on. Based on this information, a schedule was made for each village leader to indicate when the survey and physical examination would take place and who should attend each component. The village chiefs then invited towns people to a meeting where they described the study, including all tests and procedures. If villagers were interested in participating, they were instructed to schedule an appointment for their physical exam and survey.

The surveys were administered at the township hospital when the villagers came for their scheduled appointment. A free physical exam was provided to each subject as an incentive for participating in the survey and included measurement of height and weight, a general physical exam, an electrocardiogram, a chest x-ray, an abdominal B ultrasound, and blood tests including routine HIV, HCV, HBV, and syphilis tests. The physical examination took place first and was followed by an interview in a private room by a trained Yi interviewer who used a questionnaire to guide the interview. During the interview, the interviewer transcribed participant responses onto the questionnaire, and all questionnaires were collected by a quality control checker to ensure that all questions had been answered and that no logistical mistakes had been made. The questionnaires were then handed to a researcher responsible for storing the data in a special, locked file cabinet. Four students were hired and trained to input the data into the electronic database. We obtained oral consent from participants prior to participation because most participants were illiterate and unable to provide written consent. On this basis, we requested that all 11 village chiefs would organize a meeting attended by all adults before this survey was conducted. In this meeting, the village chiefs explained the survey to the participants, and the aims of the Chinese government in conducting this project. All participants were informed that this survey included a questionnaire and free physical examination, and the questionnaire included details of health-related practices and high risk behaviors, such as drug use and multiple sexual partners. The physical examination included measurements of height and weight, a general physical examination, an electrocardiogram, a chest x-ray, an abdominal B ultrasound, and blood tests including routine blood tests, HIV, HCV, HBV, and syphilis tests. Some items were not provided to individuals outside of the designated age bracket or if they were pregnant. Regarding children, if their parents agreed, they also underwent this survey at the township hospital, including the questionnaire and physical examination. At the end of the survey, each participant received 20 RMB and their fingerprint was recorded on a list of individuals in the region. All of these procedures and the survey content were approved by the institutional review board (IRB) of the Si-chuan Center for Disease Control and Prevention.

### Measures

Socio-demographic data were collected with a 29-item face-to-face interview that was designed to assess HIV related risks and pilot tested to be sensitive to the local economic and cultural situation. The questions used in this study involved three sub-sections in the questionnaire: demographic characteristics (such as name, age, gender, marital status, education and occupation, and so on), high risk behaviors (such as drug use and multiple sexual partners), and knowledge of HIV/AIDS.

All residents were provided the physical examination and blood tests. Because of the possibility that children under age 14 were infected through mother-to-child transmission and because local customs permit young people (over 14 years old and unmarried) to have multiple concurrent sexual partners, we only analyzed data from subjects over 14 years old. A sub-group analysis was conducted among single participants. Age was divided into the following groups: 14–24, 25–34, 35–44, and 45+. Because most people were illiterate, and only a few went to junior high school, we combined those that had attended primary school or higher education into one group (primary school or above). Marriage status was divided into three groups: never married, married, and divorced/widowed. Most people reported their occupations as student, farmer, or migrant worker (worked outside the town); very few people had other careers, so they were combined into the “student and other” group.

The local Yi culture allows multiple sexual partners for both young men and women before marriage, but extramarital sex is not acceptable after marriage. Due to these customs, only participants who were 14 years of age or younger and single were asked about their sexual partners in the interview. If the response to the question, “Do you have a sexual partner?” was “Yes”, the subjects would be asked follow-up questions such as “how many sexual partners do you have?”

Drug use status referred specifically to heroin use and was determined from two sources of information: 1) survey questions, such as “Is there any heroin use among your friends?”, “Have you ever used heroin?”, and 2) drug use status reported by village chiefs. Ultimately, we decided to rely on the village chiefs' report of drug use to determine individual drug use status. We found that survey questions were too sensitive due to government drug control enforcement; thus, severe reporting bias existed and very few participants reported drug use. Village chiefs were determined to be a reliable source of information because of their status and the intimacy of the local population. People in a village communicate frequently with each other and drug use status is often known by the entire community. Furthermore, the village chiefs' prestige facilitated the acquisition of additional information about individuals, and they often help the government in enforcing drug control efforts.

To determine HIV knowledge, participants were first asked “Do you know of AIDS?”, if they answered “No”, they would be coded into the “no knowledge group”, if the answer was “Yes”, they would be asked four questions about their knowledge of HIV blood transmission, including injectable drugs, sexual transmission, and pregnant mother-to-child transmission. If the participant answered “Yes,” that he or she knew of the respective type of HIV transmission to all four questions, the participant would be coded into an “all knowledge” group. If the participant did not answer all four questions correctly, he or she would be coded into a “some knowledge” group. Our survey did not include questions about HCV, so our analysis of the relationship between knowledge and HIV infection does not take into account HCV knowledge.

### Blood testing

All blood samples were transported from township hospitals to the Butuo county hospital for serum separation by laboratory technicians within 24 hours, and then transported to the Liangshan Prefecture CDC for HIV and HCV testing. HIV antibodies were screened using enzyme-linked immunosorbent assay (ELISA; Anti-HIV Antibodies ELISA Diagnostic Kit, Livzon Diagnostics Inc, China; Anthos 2010, Anthos Fluido 2, Biochrom Ltd.); if the result was positive, two additional assays (the original assay plus a different, confirmatory assay) were conducted in parallel. If both were positive or the results were discordant, a confirmatory test was done using a bolt assay (Gebelabs Diagnostics Pte Ltd., Caverdish, Singapore). An ELISA for anti-HCV IgG antibodies was conducted to determine HCV infection status (ELISA, HCV Antibodies Kit, King Hawk Pharmaceutical, Ltd.; Anthos 2010, Anthos Fluido 2, Biochrom Ltd.), according to the manufacturer's protocol. All tests were performed strictly under national operation standards. The results were filed in the participants' physical examination forms by the researchers.

### Data analysis

All data collected by paper-and-pencil surveys were inputted manually into a custom designed database and analyzed using SPSS for Windows Version 17.0 (IBM Corporation, Armonk, NY, USA). Descriptive statistics were generated for each general characteristic variable. We used Chi-square tests to compare differences between different demographic and risk groups. Adjusted and unadjusted logistic regression analysis was performed to test risk factors associated with HIV, HCV, and co-infection. All statistical tests were two-sided with a significance level of *P*<0.05.

## Results

### Demographic characteristics

Of the 10,939 residents registered with the village chiefs in the two towns, 10,104 (92.4%) participated the survey and 9,179 provided blood samples for HIV and HCV testing between April 2010 to December 2010. Since local Yi indigenous culture considers age 14 as the start of adulthood, when sexual debut and marriage are acceptable, we included only the 6,072 participants who were at least 14 years old and who completed both the survey and HIV/HCV testing. The demographic characteristics of the study participants are shown in Table 1. Participants were predominantly of Yi nationality (99.6%), nearly half (48.6%) were males, and most participants were married (80.7%), illiterate (84.4%), and worked as farmers (74.5%). Only 3.7% of participants reported seeking job opportunities outside the district.

**Table 1:** Demographic characteristics of study participants.

	N	Percentage (%)
**Age**		
14–24	1743	28.7
25–34	1330	21.9
35–44	1190	19.6
Over 45	1809	29.8
**Gender**		
Male	2954	48.6
Female	3118	51.4
**Ethnicity**		
Yi	6048	99.6
Han	21	0.3
Other	3	0.1
**Marital status**		
Never married	945	15.6
Married	4901	80.7
Divorced/widowed	213	3.5
Missing	13	0.2
**Education**		
Illiteracy	5119	84.3
Primary school or above	929	15.3
Missing	24	0.4
**Occupation**		
Students and other	140	2.3
Farmer	4511	74.5
Migrant worker	224	3.7
Missing	1197	19.7

### HIV/HCV prevalence by demographic, drug use status, and HIV knowledge

The rates of HIV, HCV, and HIV/HCV co-infection for all residents over 14 years old were 11.4%, 14.0%, and 7.7%, respectively (Table 2). Compared to other age groups, the 25–34-yr age group had the highest prevalence of HIV, HCV, and HIV/HCV co-infections, reaching rates of 24.4%, 26.2% and 16.0%, respectively. Males had a much higher prevalence of all infections compared to females (HIV: 16.3% vs. 6.8%, HCV: 24.6% vs. 3.9%, HIV/HCV co-infected: 14.7% vs. 1.1%, respectively; *P* = 0.000 for all), and married participants had higher rates of HIV, HCV, and co-infection, followed by single participants and divorced or widowed participants. Using the drug use information provided by village chiefs, we found that about half of drug users tested positive for HIV (48.7%) and more than half (68.4%) tested positive for HCV. In addition, migrant workers were more likely to test positive for HIV and HCV and co-infection than other work groups (*P*<0.01). There was no significant difference in rates of HIV, HCV, and co-infection by education level. Participants who had the least knowledge about HIV had the lowest rate of HIV infection (*P* = 0.00).

**Table 2:** Chi-square analysis of HIV and HCV prevalence in the study population.

	N	HIV (%)	HCV (%)	HIV & HCV (%)
**Total**	6072	11.4	14.0	7.7
**Age (yr)**				
14–24	1743	11.9	16.3	9.2
25–34	1330	24.4	26.2	16.0
35–44	1190	10.6	14.6	6.7
Over 45	1809	1.9	2.4	0.7
**Gender**				
Male	2954	16.3	24.6	14.7
Female	3118	6.8	3.9	1.1
**Marital status**				
Never married	945	9.3	12.4	7.4
Married	4901	12.0	14.7	7.9^*^
Divorced/widowed	213	5.6	6.1	3.3
**Education**				
Illiteracy	5119	11.5	13.6	7.5
Primary school or above	929	10.5^*^	16.5	8.7^*^
**Occupation**				
Student and others	140	6.4	11.4	6.4
Farmer	4511	11.8	14.6	8.0
Migrant worker	224	18.8	28.1	16.5
**Reported drug use**				
No	4852	7.7	8.8	4.1
Yes	158	48.7	68.4	44.9
**Knowledge of transmission types**				
All knowledge	2806	12.2	13.9	7.7
Some knowledge	1034	11.9	15.4	8.0
No knowledge	2049	8.8	11.4	5.9

Note: All differences between groups were significant (*P*<0.05), except for those labeled with *.

Abbreviations: HCV, hepatitis C virus; HIV, human immunodeficiency virus.

Bivariate and multivariate logistic regression analysis were undertaken to identify risk factors associated with HIV and HCV infection. Table 3 presents the adjusted odds ratio (AOR) and unadjusted odds ratios (UOR) for HIV, HCV, and co-infection. Significant risk factors indicated by the AOR were age group (especially 25–34 years old, AOR: 13.6, 95% [confidence interval] CI 9.4–19.7 for HIV, and AOR: 13.8, 95%CI 9.7–19.5 for HCV) and drug abuse (AOR: 6.9 for HIV and AOR: 11.8 for HCV). Other risk factors, including being male, illiterate, working as a farmer, migrant worker, divorce/widowed, were marginally significant in multivariate logistic regression models. There was no significant relationship between knowledge and HIV infection. Males were more frequently infected by HCV than by HIV; the AOR rose from 2.4 for HIV (95%CI: 2.0–2.9) to 7.8 for HCV (95%CI: 6.3–9.7).

**Table 3:** Logistic regression analysis of HIV risk factors.

	HIV OR (95%CI)	HCV OR (95%CI)	HIV AOR (95%CI)	HCV AOR (95%CI)
**Age (yr)**				
14–24	6.9 (4.9–9.9)	7.8 (5.6–10.8)	7.6 (5.1–11.3)	9.9 (6.9–14.3)
25–34	16.3 (11.4–23.3)	14.2 (10.3–19.6)	13.6 (9.4–19.7)	13.8 (9.7–19.5)
35–44	6.0 (4.1–8.8)	6.9 (4.9–9.6)	5.0 (3.4–7.5)	6.3 (4.4–9.1)
Over 45	1.0	1.0	1.0	1.0
**Gender**				
Male	2.7 (2.3–3.2)	7.9 (6.5–9.7)	2.4 (2.0–2.9)	7.8 (6.3–9.7)
Female	1.0	1.0	1.0	1.0
**Marital status**				
Never married	1.0	1.0	1.0	1.0
Married	1.3 (1.1–1.7)	1.2 (1.0–1.5)	1.6 (1.2–2.1)	2.0 (1.5–2.7)
Divorced/widowed	0.6 (0.3–1.1)	0.5 (0.3–0.8)	2.1 (1.0–4.5)	2.7 (1.2–5.8)
**Education**				
Illiteracy	1.1 (0.9–1.4)	0.8 (0.7–1.0)	1.5 (1.1–1.9)	1.4 (1.1–1.8)
Primary school or above	1.0	1.0	1.0	1.0
**Occupation**				
Student and others	1.0	1.0	1.0	1.0
Farmer	1.9 (1.0–3.8)	1.3 (0.8–2.2)	2.0 (1.0–4.1)	1.6 (0.9–2.9)
Migrant worker	3.4 (1.6–7.1)	3.0 (1.7–5.5)	1.9 (0.9–4.3)	1.7 (0.9–3.3)
**Drug abuse**				
No	1.0	1.0	1.0	1.0
Yes	11.4 (8.2–15.9)	22.3 (15.7–31.7)	6.9 (4.8–9.9)	11.8 (8.0–17.6)
**Knowledge**				
All known	1.0		1.0	
Not all known	1.0 (0.8–1.2)		1.1 (0.9–1.5)	
Unknown	0.7 (0.6–0.8)		0.8 (0.7–1.00)	

Abbreviations: CI, confidence interval; HCV, hepatitis C virus; HIV, human immunodeficiency virus; OR, odds ratio.

### Subgroup analysis: HIV/HCV prevalence among singles

Table 4 shows that HIV and HCV infection rates in the singles sub-population were 9.3% and 12.4%, respectively, both lower than the overall study population, especially among women (HIV: 6.8% overall vs. 2.6% among single women; HCV: 3.9% overall vs. 2.9% among single women). Males had a higher rate of HIV and HCV infection than females (HIV: 13.3% vs. 2.6%, HCV: 18.0% vs. 2.9%, respectively); these differences were significant (*P*<0.01). Participants who reported having multiple sexual partners had higher HIV and HCV infection rates (HIV: 13.9%, HCV: 18.9%, HIV and HCV co-infection:11.8%) than those reporting none or only one sex partner. There was no significant difference in infection rates between groups stratified by HIV knowledge. The difference in HIV and HCV infection rates by different education levels was significant (*P*<0.05; OR = 2.29). Drug use was the most important risk factor for HIV prevalence. HIV prevalence in IDUs reached 54.8%, and the rate of HCV infection was even greater, at 71.0%. We also carried out univariate logistic regression analysis, which showed that five factors were significantly associated with HIV and HCV infection: gender (OR = 5.8), education (OR = 2.29), occupation (student as reference; farmer: OR = 5.02, migrant worker: OR = 6.12), drug abuse (OR = 18.0), and multiple sexual partners (OR = 2.92; Table 4); knowledge was not significant.

**Table 4:** Chi-square and logistic regression analysis of HIV and HCV prevalence among unmarried participants over the age of 14.

	N	HIV (%)	HCV (%)	HIV/HCV (%)	OR for HIV (95%CI)	OR for HCV (95%CI)
**Total**	945	9.3	12.4	7.4		
**Gender**						
Female	350	2.6	2.9	0.6	1.0	1.0
Male	595	13.3	18.0	11.4	5.8 (2.9–11.7)	7.5 (3.8–14.5)
**Education**						
Primary school or above	419	5.7	9.8	4.5	1.0	1.0
Illiteracy	516	12.2	14.5	9.7	2.3 (1.4–3.7)	1.6 (1.0–2.4)
**Occupation**						
Student and others	81	2.5	4.9	2.5	1.0	1.0
Farmer	594	11.3	15.8	8.9	5.0 (1.2–20.9)	3.6 (1.3–10.1)
Migrant worker	82	13.4	14.6	12.2	6.1 (1.3–28.6)	3.3 (1.0–10.7)
**Knowledge**						
All known	412	9.2			1.0	
Not all known	130	13.1^*^			1.5 (0.8–2.7)	
Unknown	359	7.0			0.7 (0.4–1.3)	
**Drug use**						
No	823	6.3	8.4	4.6	1.0	1.0
Yes	31	54.8	71.0	51.6	18.0 (8.4–38.5)	26.7 (11.8–60.3)
**Sexual partners**						
None or only one	460	5.2	7.4	3.7	1.0	1.0
Multiple	238	13.9	18.9	11.8	2.9 (1.7–5.1)	2.9 (1.8–4.7)

Abbreviations: CI, confidence interval; HCV, hepatitis C virus; HIV, human immunodeficiency virus; OR, odds ratio.

## Discussion

In this study, the prevalence of HIV was 11.4% in the >14 years age group. This is the highest ever reported rate by annual surveillance performed in Liangshan Prefecture or other areas in the People's Republic of China, and far greater than the total population prevalence in the People's Republic of China (0.057%) [1]; these numbers are even higher than mean infection levels in Sub-Saharan Africa (5.0%) [15]. These numbers imply that HIV prevalence in this region is very serious, and surrounding areas should increase HIV testing efforts (we have already started extending the scope of this survey).

The results also showed that the rate of HIV infection among males (16.3%) was much higher than among females (6.8%). This finding is consistent with surveillance results in Liangshan [16] and suggests that there are greater risk factors for HIV in males than in females. Results of risk behavior analyses confirmed that men did indeed have higher rates of intravenous drug use (4.5% vs. 0.8%) and multiple sexual partners (6.9% vs. 2.1%) than women.

Our study also found high levels of HCV prevalence (14.0%) compared to worldwide estimates of HCV infection (3.3%), and the total population prevalence of HCV in the People's Republic of China [17], [18]. The rate of HCV infection among men was also higher than among women. This finding is consistent with higher HIV rates among men than women, providing evidence that HCV shares the same transmission routes as HIV [19]. However, the proportion of residents with HIV that were co-infected with HCV (62.5%) was lower than what has previously been reported with respect to HCV transmission through drug use [20]–[23]. This implies that blood transmission is not the only primary transmission route for HIV in this region, in which the HIV epidemic was historically driven by injection drug use, and that sexual transmission is now accelerating its spread. This was comparable to annual surveillance results in this region, which show that the proportion of residents infected with HCV by sexual transmission is rising. Furthermore, men had a higher risk of HIV and HCV co-infection than women. The results in Table 3 also showed that the AOR was 2.4 for HIV and 7.8 for HCV, which suggested that the greater risk of co-infection in men compared to women could be attributable to a greater risk of HCV infection. This may be due to men having high rates of needle sharing, and is consistent with results that HCV can transmit through intravenous drug use 10 times more efficiently than HIV [24].

The results of the logistic analysis showed that drug use (OR = 6.9 in the over 14 age group analysis; OR = 18.0 in unmarried participants over 14 years of age), multiple sexual partners (OR = 2.9 in unmarried participants over 14 years of age), being a migrant worker (working outside of the town, OR = 3.36), and being married (OR = 1.33) were risk factors for HIV infection. However, illiteracy and lack of knowledge did not increase the risk of HIV infection. There may three reasons for this finding. First, the education level in this region was very low: 84.3% of the total population was illiterate and few people went to school. Even those that did go to school achieved very low literacy scores. Second, many subjects were already infected when they learned about HIV. It is possible, then, that HIV risk behaviors occurred prior to acquiring knowledge of HIV. This result, however, should not undermine the importance of HIV education. Instead, our results stress the need for the government to enhance overall education in these areas. In this study, we also found that the prevalence of HIV in the 14–24-yr and 25–34-yr age groups were higher than in the other groups, at 24.4% (OR = 16.3) and 11.9% (OR = 6.9), respectively. This finding implied that individuals in these two age groups had more risk factors than those in the other groups, especially in the 25–34-yr age group. Individuals in this age bracket were found to have higher rates of drug abuse, were more sexually active and had a longer period of risk for exposure of HIV, as the first HIV infection was reported in 1995 in this region. Among all participants, men had a higher HIV infection rate than women (OR = 2.67), suggesting that men have greater risk factors for infection. A higher rate of drug use (male: 4.5%, female: 0.8%) among men confirmed this point.

Due to the local culture in this ethnic minority, we did not ask about multiple sexual partners for participants who reported that they were married; however, we have collected sufficient data from the groups that were aged over 14 years and those that were unmarried, and hope that this data will make up for this deficit. Butuo is a minority area and local customs dictate that before marriage, young people are allowed to have multiple sexual partners (for example, on special dates each month young people are allowed to freely look for sexual partners at public market gatherings), but after marriage, multiple sexual partners are strictly prohibited. As we did not ask married individuals about multiple sexual partners, our analysis here focuses only on unmarried participants. We found that HIV and HCV prevalence in the married versus unmarried groups were 9.3% and 12.4%, respectively. These are both higher than the divorced/widowed group (HIV: 5.6%, HCV: 6.1%). Combined with the results of multiple sexual partners (male: 29.9%, female: 17.1%), which were higher than in other, low HIV prevalence regions [25], we think that having multiple sexual partners is a very important risk factor for HIV infection. In contrast to the total study population, we found that education was related to HIV infection, but knowledge was not. This suggests that education, which improves comprehension, is necessary to control HIV. Only focusing on several knowledge points is not enough. Compared to the total population, we also found that the OR in the male group increased from 2.7 to 5.8 in the over 14 years old group and also in the unmarried group. This implies that this group is the most important risk population and, aside from drug use, multiple sexual partners may be more common and important in this group than other groups, and more attention should be paid to these groups when developing intervention strategies.

Our study has several limitations. Firstly, we did not collect data on multiple sexual partners in the married population because the local culture prohibits extramarital behavior after marriage. Because of this custom, very few participants were likely to acknowledge that they had had multiple sexual partners after marriage. Secondly, drug abuse data was not self-reported by participants. At the time we conducted the survey, the local government was carrying out an anti-drug project in which all drug users would be subjected to mandatory drug treatment. Therefore, many drug users did not dare say they were drug users for fear of having to undergo the mandatory drug treatment. This may have resulted in lower rates of reported drug use and multiple sexual partners in our data. Thirdly, due to the local culture, we did not ask about multiple sexual partners for participants who reported that they were married. Most study participants were unwilling to discuss sexual behaviors, especially because the study was conducted over such a short time. In order to make up for this deficiency, we will conduct separate in-depth interviews about sexual behaviors during 2014, including extramarital sex and sex within marriage. Fourthly, publication has been delayed by the length of time needed to analyze and check the raw data. Fifth, Liangshan includes 17 counties while the vast majority of Yi minority live in 8 counties, which are Zhaojue, Butuo, Puge, Xide, Meigu, Yuexi, Jinyang and Ganluo. These eight counties are adjacent to each other and share a great amount of similar cultural characteristics and social traditions. Therefore, in this study we only choosed two towns for a population-based survey, we know this can not represent the whole minority region of Liangshan. and hope we can expand the scope of the study in the future.

In summary, we believe that drug use, multiple sexual partners, and low education levels were the three main risk factors leading to HIV prevalence in this region. The government should pay more attention to improving education and personal health consciousness while enhancing drug control programs and changing bad habits, as well as unhealthy minority customs.
